# High-Intensity Exercise Performance as a Complex Adaptive System: An Integrative Review

**DOI:** 10.3390/muscles5030053

**Published:** 2026-07-24

**Authors:** Walaa Jumah Alkasasbeh, Adam Tawfiq Amawi, Gerasimos V. Grivas

**Affiliations:** 1 Department of Physical Education, School of Sports Sciences, The University of Jordan, Amman 11942, Jordan; 2 Department of Movement Sciences and Sports Training, School of Sports Science, The University of Jordan, Amman 11942, Jordan; a.amawi@ju.edu.jo; 3 Physical Education and Sports, Division of Humanities and Political Sciences, Hellenic Naval Academy, 18539 Piraeus, Greece; grivasger@hotmail.com

**Keywords:** high-intensity exercise, neuromuscular adaptations, mitochondrial function, metabolic stress, mechanotransduction, ergogenic aids, performance optimization

## Abstract

High-intensity exercise performance is a complex phenomenon that emerges from the interaction of neuromuscular, metabolic, molecular, and biomechanical systems. While previous research has primarily examined these factors independently, a comprehensive understanding requires an integrative perspective. Therefore, this narrative review aimed to synthesize current evidence and conceptualize high-intensity exercise performance as a complex adaptive system. The review examined the effects of different training modalities, including high-intensity interval training, sprint training, resistance training, plyometric training, and concurrent training, alongside neuromuscular regulation, molecular adaptations, fatigue mechanisms, and ergogenic aids. Evidence indicates that performance improvements result from coordinated interactions among training-induced adaptations, intracellular signaling pathways, and physiological responses. Furthermore, ergogenic aids such as caffeine, creatine, beta-alanine, and dietary nitrates may enhance performance when appropriately aligned with training demands. Overall, high-intensity exercise performance should be viewed as an emergent property of interconnected physiological systems rather than the product of isolated mechanisms. This systems-based framework provides a comprehensive perspective for understanding and optimizing athletic performance.

## 1. Introduction

High-intensity exercise performance is a complex and multifactorial construct that arises from the interaction of physiological, neuromuscular, metabolic, and psychological systems rather than a single determinant [1,2,3,4,5]. Existing literature highlights that performance in high-intensity activities depends on the coordinated contribution of aerobic and anaerobic energy systems, as well as the efficiency of fatigue resistance and recovery processes [1,6,7,8]. In this context, fatigue is considered a primary limiting factor, influencing performance through both physiological and neuromuscular mechanisms, including energy depletion, metabolite accumulation, and reduced muscle activation [1,9,10].

Training modalities play a crucial role in enhancing high-intensity performance, with evidence supporting the effectiveness of approaches such as HIIT and combined training, particularly when integrated with nutritional and behavioral factors [11,12,13,14,15]. In particular, HIIT has been shown to induce significant improvements in cardiovascular fitness, metabolic efficiency, and performance outcomes, especially when combined with appropriate nutritional strategies and ergogenic aids [16,17,18]. Furthermore, the integration of resistance-based and plyometric stimuli has been suggested to optimize strength, power, and neuromuscular performance beyond the effects of isolated training modalities [2,19,20].

At the neuromuscular level, adaptations to high-intensity training include enhanced motor unit recruitment, increased firing frequency, and improved synchronization, which collectively contribute to improvements in strength, power, and rate of force development [2,21,22]. These adaptations are essential for explosive movements and high-intensity efforts requiring rapid force production. However, it has been suggested that HIIT alone may not be sufficient to maximize maximal strength and hypertrophy without the inclusion of progressive overload through resistance training [2].

From a physiological perspective, repeated exposure to high-intensity exercise induces substantial adaptations in energy metabolism, including improvements in oxygen utilization, metabolic efficiency, and overall cardiorespiratory function [1,11,18]. These adaptations highlight the integrative role of aerobic and anaerobic metabolism even during short-duration, high-intensity efforts [1,17]. Additionally, performance is strongly influenced by the ability to tolerate and manage fatigue, which is associated with metabolic stress, acid–base balance, and neuromuscular factors [1,10,23].

At the molecular level, exercise activates intracellular signaling pathways, with reactive oxygen species (ROS) acting as key regulators of AMPK, MAPK, NRF2, and PGC-1α, which drive mitochondrial biogenesis, glucose uptake, and muscle hypertrophy [23]. However, excessive antioxidant supplementation may impair these beneficial adaptations by disrupting ROS-mediated signaling, emphasizing the importance of maintaining redox balance [23]. Skeletal muscle exhibits remarkable plasticity, with long-term training adaptations driven by repeated exercise-induced signaling that integrates neural, mechanical, metabolic, and hormonal stimuli, alongside epigenetic and post-transcriptional regulation [24].

In addition to physiological and molecular mechanisms, ergogenic aids, particularly nutritional strategies and supplementation, have been widely investigated for their potential to enhance high-intensity performance [7,13,15,16].

While some ergogenic aids may improve performance outcomes, their effectiveness often depends on individual characteristics, training status, and the specific demands of the activity [12,14,19].

Traditional sport science has often relied on reductionist models that overlook the dynamic, multi-level interactions underlying performance, whereas contemporary complex systems perspectives emphasize integration, adaptability, and synergy across molecular, physiological, psychological, and environmental factors [25]. However, a persistent research–practice gap remains due to fragmented approaches and limited ecological validity, underscoring the need for integrative, multidisciplinary frameworks to bridge theory and applied practice [26].

Although substantial progress has been made in understanding the individual determinants of high-intensity exercise performance, the existing literature remains predominantly reductionist. Most studies and reviews have examined isolated domains such as neuromuscular function, metabolic adaptations, fatigue mechanisms, molecular signaling, resistance training, HIIT, or ergogenic aids independently. While these reductionist approaches have generated important mechanistic insights, they provide only a partial explanation of performance because they overlook the dynamic interactions, reciprocal feedback loops, and context-dependent adaptations that emerge across multiple physiological systems during real-world exercise.

This limitation has become increasingly apparent with the emergence of systems biology, Complex Adaptive Systems theory, and network physiology, which collectively recognize biological function as an emergent property arising from continuous interactions among interconnected systems rather than from isolated mechanisms. Despite these conceptual advances, the current literature remains fragmented across disciplinary boundaries, with no previous narrative review comprehensively integrating training modalities, neuromuscular regulation, biomechanical loading, mechanotransduction, molecular signaling, metabolic stress, fatigue regulation, and ergogenic interventions within a unified multilevel framework of high-intensity exercise performance. Consequently, the translation of mechanistic evidence into ecologically valid and practically applicable training models remains limited.

The central research question guiding this review is: How can high-intensity exercise performance be more comprehensively understood by conceptualizing it as a Complex Adaptive System rather than as the sum of isolated physiological mechanisms? Although previous studies have generated substantial knowledge regarding individual physiological determinants of performance, no narrative review has explicitly synthesized these domains within a unified Complex Adaptive Systems framework capable of explaining their dynamic interactions, reciprocal feedback mechanisms, and context-dependent adaptations. Addressing this conceptual gap provides the rationale for the present review.

Accordingly, this narrative review develops an integrative conceptual framework that reconceptualizes high-intensity exercise performance as a Complex Adaptive System, in which performance emerges from dynamic, nonlinear, and reciprocal interactions among multiple physiological systems. Rather than providing exhaustive standalone reviews of individual physiological domains, the selected evidence is synthesized to demonstrate how each domain functions as a critical subsystem within an integrated systems-level framework. The principal contribution of this review lies not in describing individual physiological mechanisms, but in explaining how their dynamic interactions collectively generate context-dependent performance adaptation. Specifically, this review sought to: (1) synthesize evidence across multiple organizational and physiological levels; (2) explain the reciprocal interactions linking training stimuli, neuromuscular regulation, mechanotransduction, molecular signaling, metabolic stress, fatigue regulation, and ergogenic modulation; (3) demonstrate how these multilevel interactions generate nonlinear and context-dependent performance adaptations; and (4) provide a systems-based conceptual framework capable of guiding future mechanistic research and informing individualized, context-specific, evidence-based training and nutritional strategies.

## 2. Methodological Approach

This study was conducted as an integrative narrative review and prepared in accordance with the Scale for the Assessment of Narrative Review Articles (SANRA), which provides methodological guidance for conducting and reporting high-quality narrative reviews [27]. The review aimed to synthesize and critically interpret contemporary evidence on the physiological determinants of high-intensity exercise performance and to develop an integrative conceptual framework based on the principles of Complex Adaptive Systems (CAS) and Network Physiology.

A targeted literature search was conducted in PubMed, Scopus, Web of Science, and Google Scholar to identify peer-reviewed studies published in English through March 2026. The search strategy combined controlled vocabulary and free-text terms related to high-intensity exercise and systems physiology, including “*high-intensity exercise*,” “*high-intensity interval training*,” “*HIIT*,” “*sprint training*,” “*resistance training*,” and “*plyometric training*,” together with terms such as “*neuromuscular adaptation*,” “*fatigue*,” “*mechanotransduction*,” “*molecular signaling*,” “*mitochondrial adaptation*,” “*ergogenic aids*,” “*complex adaptive systems*,” “*systems biology*,” and “*network physiology*”. The reference lists of key studies and relevant review articles were also manually screened to identify additional influential publications.

Because the primary objective of this review was conceptual integration rather than exhaustive evidence retrieval, studies were selected using a purposive selection approach. Eligible publications included peer-reviewed original research articles, mechanistic studies, and high-quality narrative or systematic reviews that provided direct evidence on physiological mechanisms, training adaptations, or systems-level interactions relevant to high-intensity exercise performance. Conference abstracts, editorials, non-peer-reviewed publications, duplicate records, and studies that were not relevant to the objectives of this review were excluded.

The screening process involved an initial review of titles and abstracts, followed by full-text evaluation of potentially relevant studies. Study selection was guided by four predefined criteria: (1) conceptual relevance to the proposed Complex Adaptive Systems framework; (2) scientific significance, reflected by the study’s contribution to advancing current understanding of the physiology of high-intensity exercise performance; (3) methodological rigor, which was assessed qualitatively based on the appropriateness of the study design, methodological transparency, clarity of reporting, and consistency of the findings with the available scientific evidence; and (4) relevance to one or more of the review domains, including training modalities, neuromuscular regulation, mechanotransduction, molecular and cellular adaptation, metabolic stress, fatigue regulation, and ergogenic interventions.

Finally, the selected evidence was synthesized through qualitative critical interpretation rather than quantitative data pooling. Findings were iteratively integrated across physiological domains to identify reciprocal interactions, feedback mechanisms, nonlinear relationships, and context-dependent adaptations. This iterative synthesis informed the development of the proposed conceptual framework, which reconceptualizes high-intensity exercise performance as an emergent property arising from dynamic interactions among multiple interconnected physiological systems.

## 3. High-Intensity Exercise Performance as a Complex Adaptive System

**Figure 1:**
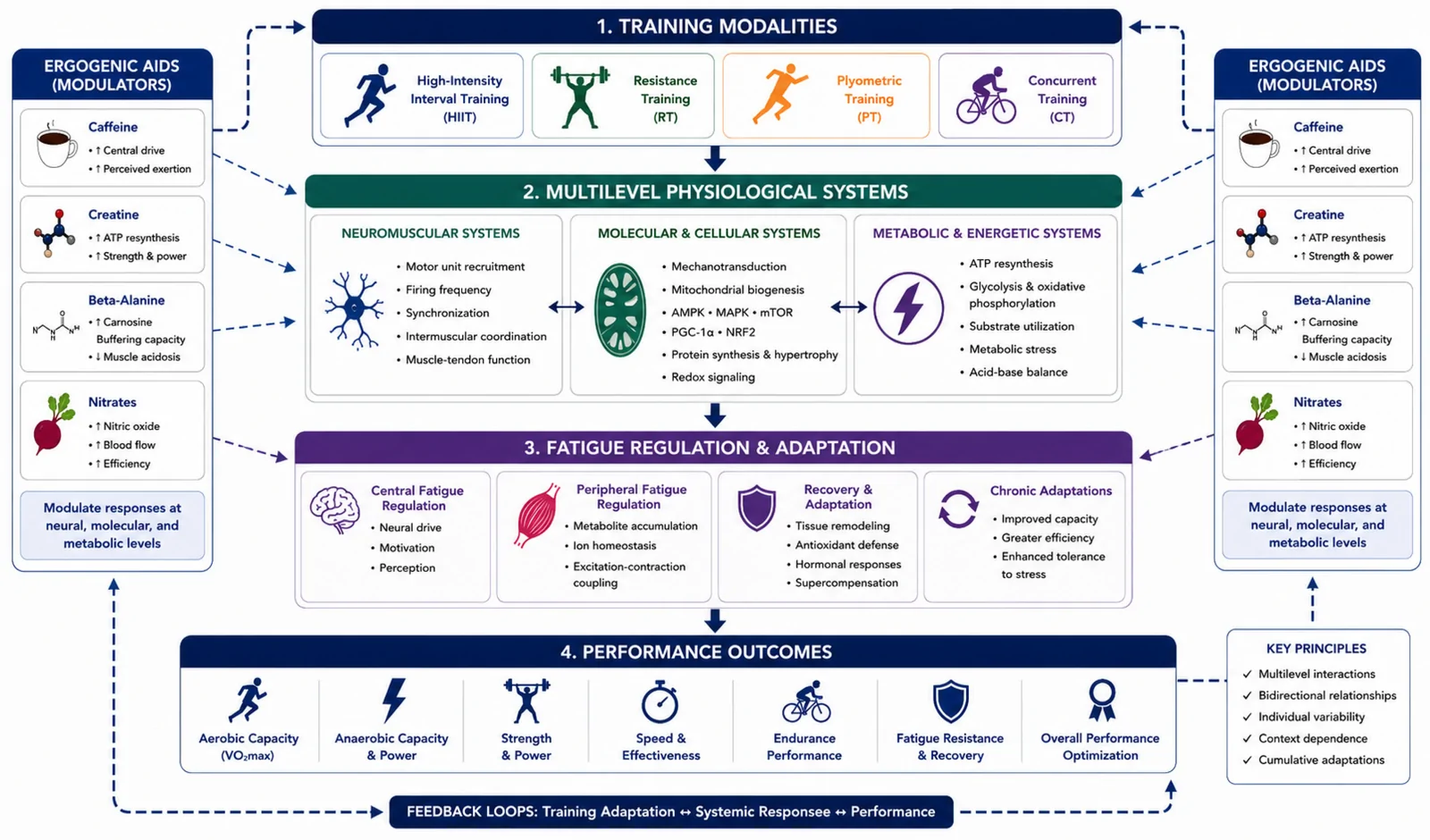
Integrated Multilevel Framework of High-Intensity Exercise Performance Adaptation.

High-intensity exercise performance is best understood as a multidimensional and dynamic construct that emerges from the interaction of multiple physiological systems rather than being governed by a single determinant [28,29]. Specifically, performance outcomes arise from the integrated contribution of neuromuscular function, bioenergetic capacity, and biomechanical efficiency, which operate in a coordinated and interdependent manner [30,31,32]. Within this framework, the neuromuscular system plays a central role in regulating force production through mechanisms such as neural drive [33], motor unit recruitment [34], firing frequency [35], and intermuscular coordination [30]. These processes determine the capacity to generate rapid and powerful movements, particularly under high-intensity conditions [2]. Simultaneously, bioenergetic processes support performance by ensuring adequate ATP availability, regulating metabolic pathways, and enhancing fatigue resistance and recovery efficiency [36,37]. In parallel, biomechanical factors influence how force is produced, transmitted, and optimized through mechanical load, force–velocity relationships, and movement efficiency [38,39]. Importantly, these systems do not function independently but interact continuously within a dynamic system characterized by bidirectional relationships and feedback loops [40]. For example, neuromuscular activation influences metabolic demand, while metabolic fatigue can modulate neural output and motor unit behavior [41,42]. Likewise, biomechanical loading conditions directly affect both neuromuscular recruitment patterns and intracellular signaling pathways associated with adaptation [35]. Therefore, performance should be viewed as an emergent property of a complex adaptive system rather than the result of isolated physiological mechanisms [43]. Training modalities act as primary drivers of this system, eliciting specific and overlapping adaptations across multiple levels [25]. High-intensity interval training (HIIT) primarily enhances metabolic efficiency and mitochondrial function, while resistance and plyometric training target neuromuscular and biomechanical adaptations, including strength, power, and rate of force development [2,44,45]. When strategically combined, these modalities can produce synergistic effects that extend beyond the outcomes of isolated training approaches [25,46,47,48]. In addition to training stimuli, ergogenic aids serve as modulators of this integrated system by influencing specific physiological pathways [49]. For instance, caffeine may enhance neural drive and reduce perceived exertion, creatine supports rapid ATP resynthesis, beta-alanine improves buffering capacity, and nitrates enhance oxygen utilization [50,51,52]. However, the effectiveness of these interventions is context-dependent and influenced by individual characteristics, training status, and the specific demands of the activity [52,53,54]. Figure 1 integrates these interacting domains into a unified conceptual framework, illustrating how reciprocal, nonlinear, and multilevel interactions collectively generate context-dependent performance adaptation rather than representing independent physiological pathways.

A defining characteristic of complex adaptive systems is nonlinearity, whereby similar training stimuli may produce markedly different physiological and performance outcomes depending on the current state of the athlete [55]. For example, a modest increase in high-intensity training load may enhance neuromuscular efficiency, mitochondrial signaling, and performance when adequate recovery and nutritional support are available [2,4,56]. However, the same increase in training load may trigger maladaptive responses, including excessive metabolic stress, autonomic imbalance, impaired motor unit recruitment, and progressive performance decline when recovery capacity is exceeded [57,58,59]. This transition illustrates that adaptation is governed by threshold-dependent interactions rather than simple cause-and-effect relationships [60,61,62]. Moreover, performance emerges from the continuous interaction of neural regulation, bioenergetic processes, and biomechanical function [63]. During repeated high-intensity exercise, increased neural drive recruits additional motor units, thereby elevating ATP demand and metabolic stress [35,64]. The resulting accumulation of metabolites modifies afferent feedback to the central nervous system, alters motor output and movement mechanics, and subsequently influences mechanical loading and intracellular signaling pathways involved in adaptation [35,65,66]. Consequently, high-intensity exercise performance cannot be explained by isolated physiological systems but represents an emergent property arising from the coordinated behavior of an interconnected biological network.

### What Does a Complex Adaptive Systems Perspective Add Beyond Conventional Exercise Physiology?

Conventional exercise physiology has traditionally explained performance by examining individual physiological systems independently, such as neuromuscular function, metabolic pathways, or molecular signaling [67,68]. While this reductionist approach has generated important mechanistic knowledge, it cannot fully explain why similar training interventions often produce markedly different outcomes between athletes or why performance frequently changes in nonlinear and context-dependent ways. A Complex Adaptive Systems (CAS) perspective extends conventional physiology by emphasizing that performance emerges from the continuous interaction of multiple interconnected physiological systems rather than from the isolated contribution of any single mechanism [69,70].

One of the principal advantages of the CAS framework is its ability to explain individual variability in training responses. Athletes exposed to identical training loads may demonstrate substantially different physiological adaptations because the response depends on the dynamic interaction between neuromuscular readiness, metabolic state, recovery capacity, nutritional status, previous training history, and other contextual factors [58,71,72]. Consequently, performance adaptation cannot be understood solely as a direct consequence of the prescribed training stimulus.

The CAS perspective also provides a theoretical basis for understanding nonlinear adaptation [73,74]. Physiological responses are not proportional to training load; relatively small changes in workload, recovery, or nutritional support may produce disproportionately large improvements or maladaptive responses once critical physiological thresholds are crossed [71,75,76]. Such threshold-dependent behavior cannot be adequately described by simple linear cause-and-effect models.

Furthermore, CAS conceptualizes fatigue as an emergent systems-level phenomenon rather than the consequence of isolated central or peripheral mechanisms [77]. Fatigue develops through reciprocal interactions among neural regulation, metabolic stress, afferent feedback, biomechanical adjustments, and intracellular signaling [78]. These continuously interacting processes dynamically influence subsequent motor output, movement efficiency, and adaptive responses, illustrating how fatigue both constrains and regulates performance.

Finally, viewing high-intensity exercise performance as a Complex Adaptive System has important practical implications for training prescription. Rather than attempting to maximize isolated physiological variables, practitioners should optimize the interactions among training load, recovery, nutrition, neuromuscular readiness, and biomechanical efficiency. This systems-based perspective supports context-specific and individualized decision-making, providing a conceptual foundation for designing adaptive training strategies that better reflect the complexity of real-world athletic performance.

Collectively, these characteristics distinguish the Complex Adaptive Systems perspective from conventional exercise physiology by emphasizing system-wide interactions, nonlinear adaptation, and context-dependent regulation. Together, they provide a more comprehensive framework for understanding, evaluating, and prescribing high-intensity exercise performance. The principal conceptual differences between these two perspectives are summarized in Table 1.

Figure 1 summarizes the conceptual framework proposed in this review. Unlike conventional models that primarily describe isolated physiological pathways, this framework emphasizes reciprocal interactions, nonlinear adaptation, and system-wide integration as the basis of high-intensity exercise performance.

## 4. Training-Induced Adaptations in High-Intensity Exercise Performance

### 4.1. Optimizing Metabolic Capacity Through High-Intensity Interval and Sprint Training

From a Complex Adaptive Systems (CAS) perspective, high-intensity interval training (HIIT) and sprint-based training should be viewed as integrated training stimuli that simultaneously influence multiple interconnected physiological systems rather than targeting metabolic function alone. Performance adaptation emerges through continuous interactions among metabolic, neuromuscular, biomechanical, and molecular processes, with reciprocal feedback loops shaping the overall adaptive response.

High-intensity interval training (HIIT) and sprint-based training are widely recognized as potent stimuli for enhancing high-intensity exercise performance through substantial bioenergetic and metabolic adaptations [2,79,80]. One of the primary adaptations associated with HIIT is the stimulation of mitochondrial biogenesis [81], leading to improved oxidative capacity [82], metabolic efficiency [83], and ATP resynthesis during repeated high-intensity efforts [36]. These mitochondrial adaptations enhance the ability to sustain performance under conditions of high metabolic demand and contribute to improved recovery between exercise bouts.

Rather than acting exclusively on metabolic pathways, HIIT simultaneously influences neuromuscular activation [2], bioenergetic regulation [84], biomechanical efficiency [85], and molecular signaling through reciprocal feedback interactions [86]. Consequently, metabolic adaptations influence subsequent neural recruitment, fatigue regulation, and movement efficiency, illustrating how system-wide interactions collectively shape performance adaptation.

In addition to oxidative adaptations, HIIT plays a critical role in improving lactate tolerance and buffering capacity [87,88,89]. Repeated exposure to high-intensity efforts promotes adaptations in acid–base regulation, allowing athletes to better tolerate metabolite accumulation and delay the onset of fatigue [4]. These metabolic responses also modify afferent feedback to the central nervous system and influence motor unit recruitment, further reinforcing the reciprocal interactions between metabolic and neuromuscular systems. This is particularly relevant in activities characterized by repeated bouts of intense effort, where the ability to sustain performance despite increasing metabolic stress is essential.

**Figure 2:**
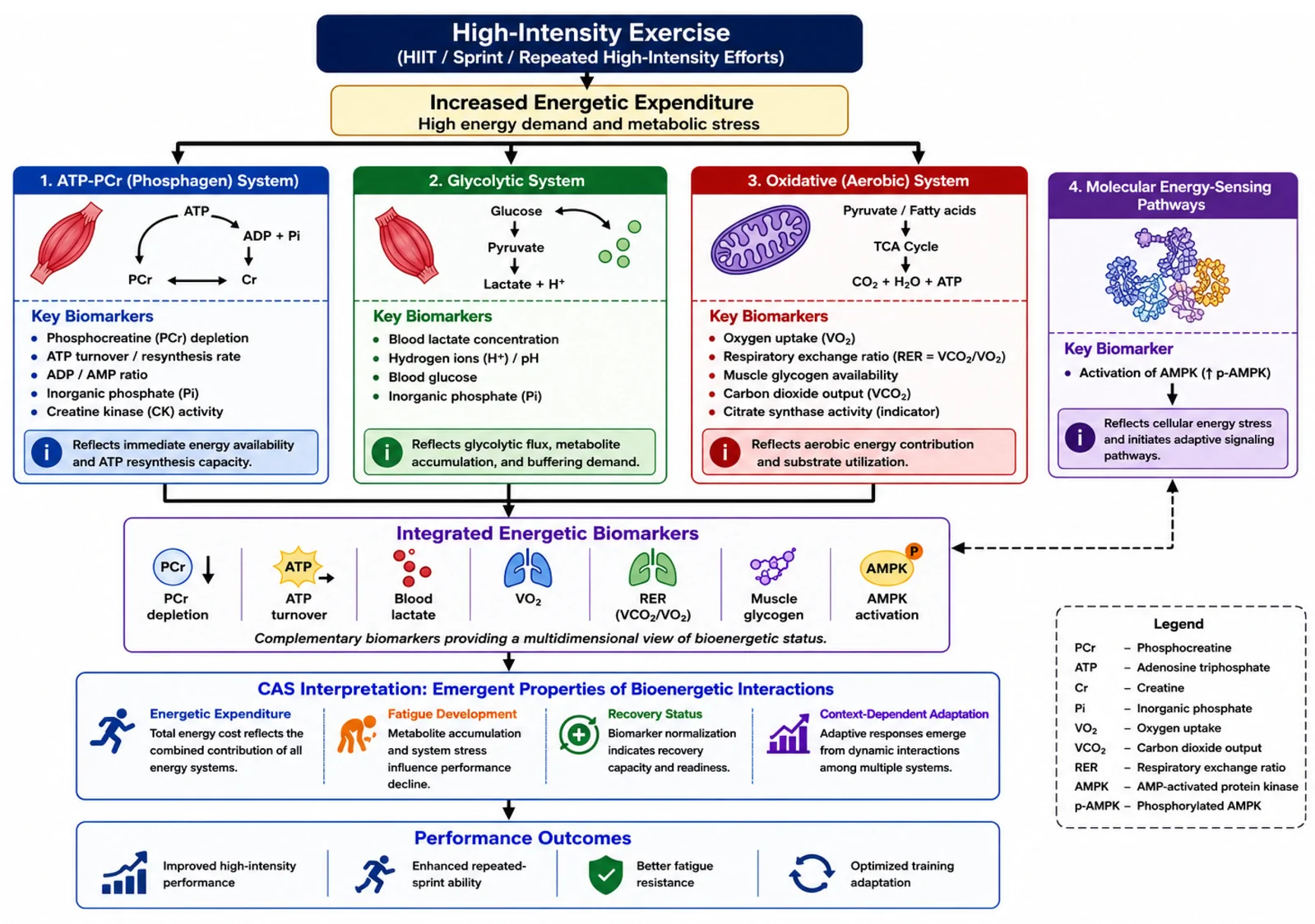
Integrated Biomarkers of Energetic Expenditure During High-Intensity Exercise.

Furthermore, sprint training and repeated sprint protocols enhance repeated sprint ability (RSA), a key determinant of performance in many team and combat sports [90,91]. These improvements are mediated by both metabolic and neuromuscular adaptations, including enhanced phosphocreatine resynthesis, improved glycolytic efficiency, and increased motor unit recruitment during high-intensity efforts [2,92]. From a systems perspective, repeated sprint ability emerges from coordinated interactions among phosphocreatine availability, metabolic regulation, neuromuscular recruitment, biomechanical efficiency, and recovery processes, rather than from improvements in any single physiological subsystem. Collectively, HIIT and sprint training target the bioenergetic dimension of performance while also contributing to neuromuscular efficiency under fatigue conditions [93,94]. From a Complex Adaptive Systems perspective, the assessment of energetic expenditure should not rely on a single physiological variable but rather on an integrated set of biomarkers that collectively reflect the athlete’s bioenergetic state. During high-intensity exercise, biomarkers such as phosphocreatine (PCr) depletion, ATP turnover, blood lactate concentration, oxygen uptake (VO_2_), respiratory exchange ratio (RER), and muscle glycogen availability provide complementary information regarding the relative contribution of the phosphagen, glycolytic, and oxidative energy systems [95,96,97,98]. At the molecular level, activation of energy-sensing pathways, including AMP-activated protein kinase (AMPK), further reflects the cellular response to energetic stress and contributes to subsequent adaptive signaling [99,100]. Importantly, these biomarkers should not be interpreted independently, as their dynamic interactions collectively characterize energetic expenditure, fatigue development, recovery status, and the context-dependent adaptation to repeated high-intensity exercise. Figure 2 illustrates how multiple biomarkers interact across phosphagen, glycolytic, oxidative, and molecular pathways to collectively characterize energetic expenditure and adaptive responses during high-intensity exercise from a Complex Adaptive Systems perspective.

However, the effectiveness of HIIT and sprint-based training is highly dependent on factors such as training status, exercise duration, and recovery intervals. While these modalities are particularly effective for improving metabolic efficiency and repeated-effort capacity, their impact on maximal strength and hypertrophy may be limited when applied in isolation. Therefore, their application should be carefully aligned with the specific performance demands and integrated with complementary training stimuli.

The adaptive responses to HIIT and sprint training remain inherently nonlinear and highly individualized [35,65,66]. Identical training stimuli may produce markedly different adaptive outcomes depending on the athlete’s current physiological state, recovery capacity, nutritional status, and training history [101,102]. Therefore, improvements in repeated high-intensity performance should be understood as emergent properties of coordinated interactions among multiple interconnected physiological systems rather than as isolated enhancements in metabolic capacity alone.

### 4.2. Enhancing Neuromuscular Function and Mechanical Efficiency Through Resistance and Plyometric Training

From a Complex Adaptive Systems (CAS) perspective, resistance and plyometric training should be viewed as integrated training stimuli that simultaneously influence neural, biomechanical, metabolic, and molecular subsystems rather than targeting neuromuscular performance alone. Adaptation emerges from dynamic interactions among these interconnected systems, with reciprocal feedback loops continuously shaping force production, movement efficiency, and long-term performance outcomes.

Resistance and plyometric training primarily target neuromuscular and biomechanical adaptations that underpin force production, power output, and movement efficiency [103]. Resistance training induces significant improvements in maximal strength through both neural and morphological adaptations [104]. Early-phase adaptations are largely driven by enhanced neural activation, including increased motor unit recruitment [105], firing frequency [106], and intermuscular coordination [101], while long-term adaptations involve muscle hypertrophy and structural remodeling [107]. Rather than acting exclusively through neural or muscular pathways, resistance training simultaneously influences biomechanical loading, mechanotransduction, metabolic regulation, and intracellular signaling, creating reciprocal interactions that collectively determine the magnitude and direction of adaptation [24,35].

Plyometric training, on the other hand, is particularly effective in enhancing the rate of force development (RFD) and explosive power through improvements in stretch–shortening cycle efficiency and neuromuscular reactivity [108,109]. These adaptations are critical for high-intensity movements requiring rapid force production, such as sprinting, jumping, and change-of-direction tasks [110]. These neuromuscular adaptations also modify mechanical loading patterns, which subsequently influence mechanotransductive signaling, tissue remodeling, and future adaptive responses, illustrating continuous feedback between neural and molecular systems.

In addition to strength and power, resistance and plyometric training improve neuromuscular efficiency, allowing for more effective force production with reduced energy cost [2,103,111]. Biomechanically, these adaptations optimize force transmission, improve movement mechanics, and enhance the interaction between muscle–tendon units [112,113]. Moreover, mechanical loading associated with resistance training activates intracellular signaling pathways that regulate muscle hypertrophy [113], protein synthesis [114], and structural adaptation [24], further contributing to performance enhancement [113]. From a systems perspective, improvements in force production arise from coordinated interactions among neuromuscular activation, biomechanical efficiency, mechanotransductive signaling, metabolic regulation, and recovery processes rather than from isolated improvements in muscular strength alone.

However, despite their well-established benefits for strength and power development, resistance and plyometric training may not sufficiently target metabolic efficiency or fatigue resistance in prolonged or repeated high-intensity efforts. As such, relying solely on these modalities may result in incomplete performance adaptations, particularly in sports requiring sustained or repeated high-intensity output. These adaptive responses therefore involve important trade-offs, as maximizing neuromuscular performance does not necessarily optimize metabolic capacity or fatigue resistance. This highlights the importance of integrating neuromuscular-focused training with metabolic conditioning strategies.

The adaptive responses to resistance and plyometric training remain inherently nonlinear and highly individualized. The magnitude and direction of these adaptations depend on the athlete’s physiological state, accumulated training load, recovery status, nutritional support, and previous training history [115]. Mechanical and metabolic signals converge on mechanotransductive signaling networks to regulate gene expression and cellular adaptation [116]. While reciprocal feedback interactions continuously reshape subsequent physiological responses. Consequently, improvements in strength, power, and movement efficiency should be viewed as emergent properties of coordinated multilevel interactions among neural, mechanical, metabolic, and molecular systems rather than the result of isolated muscular or biomechanical changes.

### 4.3. Integrating Training Stimuli for Comprehensive Performance Development

From a Complex Adaptive Systems (CAS) perspective, integrated training should be viewed as the coordinated interaction of multiple training stimuli that simultaneously influence molecular, metabolic, neuromuscular, and biomechanical subsystems. Rather than producing isolated physiological adaptations, concurrent training generates system-wide responses through reciprocal interactions, feedback loops, and context-dependent regulation.

While individual training modalities elicit specific adaptations, emerging evidence highlights the importance of integrating multiple training approaches to maximize performance outcomes [117]. Combined training models, particularly concurrent training, such as the integration of endurance or high-intensity interval training (HIIT) with resistance training, have been shown to produce complementary and, in many cases, synergistic effects that extend beyond the outcomes of isolated training modalities [45,118]. Traditionally, concurrent training was associated with the “interference effect”, where endurance training was thought to attenuate strength and hypertrophy adaptations [119,120]. However, recent evidence suggests that when appropriately designed considering factors such as training intensity, volume, sequencing, and recovery combined training can enhance both neuromuscular and metabolic adaptations simultaneously [2,101,121,122]. For example, concurrent training has been shown to improve both maximal oxygen uptake and muscular power, as well as enhance overall athletic performance [119,123,124].

From a systems perspective, integrated training approaches facilitate interactions across multiple physiological levels, including molecular, metabolic, neuromuscular, and biomechanical domains [25,125]. This integration promotes the development of functional synergies, where adaptations in one system support and enhance adaptations in others [25]. As a result, performance improvements emerge not from isolated adaptations but from the coordinated interaction of multiple systems.

At the cellular level, these interactions are coordinated through dynamic signaling networks that function as regulatory decision nodes within the Complex Adaptive System [126,127]. In particular, the reciprocal crosstalk between AMP-activated protein kinase (AMPK), which is activated by metabolic stress and energy depletion, and the mechanistic target of rapamycin (mTOR), which is stimulated by mechanical loading and amino acid availability, influences the direction and magnitude of training adaptation [59,128]. Rather than acting independently, these pathways continuously integrate metabolic, mechanical, and nutritional signals to regulate mitochondrial biogenesis, protein synthesis, and muscle remodeling according to the prevailing physiological environment [129,130,131]. Consequently, the interference effect should be viewed as an emergent systems-level response arising from dynamic interactions among multiple signaling networks rather than as the consequence of simple competition between endurance and resistance training.

Importantly, no single training modality is sufficient in isolation to fully optimize high-intensity performance [45]. Rather, the strategic integration of training modalities is essential to target the multidimensional nature of performance and to maximize adaptive potential across physiological systems [132].

However, the effectiveness of combined training models is highly dependent on the appropriate manipulation of key programming variables, including training sequence, volume distribution, and recovery strategies. Inadequate design may still result in interference effects or suboptimal adaptations, particularly when competing stimuli are not properly balanced. Therefore, the successful implementation of concurrent and integrated training approaches requires a context-specific, individualized, and periodized strategy. These adaptive responses also involve important trade-offs, as maximizing one physiological adaptation may attenuate or modify another depending on training sequence, volume, recovery, and nutritional support.

## 5. Central and Peripheral Mechanisms Regulating High-Intensity Performance

From a Complex Adaptive Systems perspective, high-intensity performance emerges from the continuous interaction of neural regulation, bioenergetic processes, and biomechanical function rather than from the independent contribution of any single physiological system. Neuromuscular regulation should therefore be understood as the product of continuous interactions among central neural drive, spinal modulation, peripheral muscle function, metabolic regulation, and biomechanical feedback.

Neuromuscular performance during high-intensity exercise is governed by complex interactions between central and peripheral mechanisms that regulate force production, coordination, and fatigue resistance [2,133]. At the central level, neural drive originating from the motor cortex plays a fundamental role in determining motor unit recruitment [134], firing frequency [41], and synchronization [135]. These processes are essential for generating rapid and powerful contractions required in high-intensity efforts [136]. Increased corticospinal excitability and improved voluntary activation following training contribute to enhanced motor output and performance capacity [137]. Rather than acting in isolation, central neural activation continuously interacts with metabolic stress, afferent feedback, and biomechanical demands, allowing motor output to adapt dynamically according to the athlete’s physiological state and exercise context.

**Figure 3:**
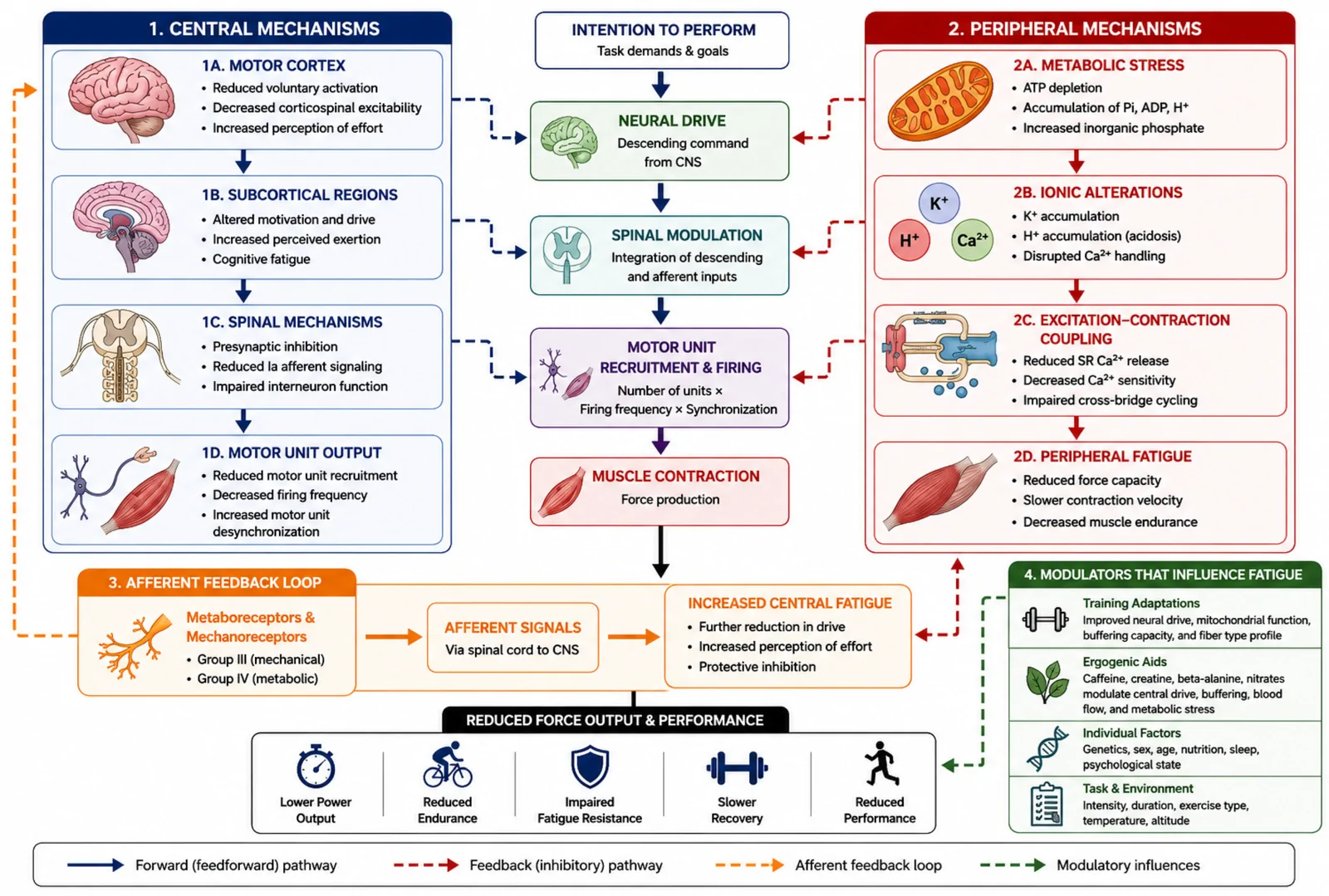
Integrated Neuromuscular Fatigue Model During High-Intensity Exercise.

As illustrated in Figure 3, neuromuscular performance is regulated through a hierarchical system involving central, spinal, and peripheral mechanisms. Neural signals originating from the motor cortex are transmitted through descending pathways to the spinal cord, where they are modulated before reaching the muscle. This multilevel organization allows for continuous adjustment of motor output based on both internal and external stimuli, ensuring optimal performance under varying conditions.

At the spinal level, α-motoneuron excitability and reflex modulation play critical roles in shaping motor output [138,139]. Afferent feedback, particularly from group III and IV afferents, provides information related to metabolic stress and mechanical strain within the muscle [140]. This feedback can exert inhibitory effects on central motor drive, contributing to the development of central fatigue during prolonged or high-intensity exercise [141]. At the same time, training adaptations may enhance the efficiency of spinal integration, improving the balance between excitatory and inhibitory inputs [142]. These spinal adaptations also influence subsequent motor unit recruitment and mechanical loading, creating reciprocal feedback loops between central neural regulation and peripheral physiological responses.

At the peripheral level, motor unit behavior is a key determinant of muscle performance [143]. The size principle governs motor unit recruitment, with smaller, fatigue-resistant units activated first, followed by larger, high-threshold motor units as force demands increase [34]. Additionally, firing rate and synchronization of motor units influence the magnitude and timing of force production [144]. These mechanisms are particularly important in high-intensity activities that require rapid force generation and precise coordination [136]. From a systems perspective, motor unit behavior both influences and is influenced by metabolic stress, afferent signaling, and movement mechanics, illustrating the bidirectional interactions that characterize neuromuscular regulation during high-intensity exercise.

Peripheral fatigue arises from multiple factors, including metabolic stress, ion imbalances, and impairments in excitation–contraction coupling [145]. Accumulation of metabolites, such as hydrogen ions and inorganic phosphate, can disrupt muscle contractility, while disturbances in calcium handling and membrane excitability further reduce force output [146]. These changes not only impair muscle function but also feed back to the central nervous system, influencing neural drive and contributing to fatigue regulation. Consequently, fatigue should not be interpreted as the failure of an isolated physiological component but as an emergent systems-level response arising from continuous interactions among neural, metabolic, and mechanical processes.

Importantly, neuromuscular fatigue should not be viewed as an isolated peripheral phenomenon but rather as an integrated response involving both central and peripheral mechanisms [147]. The interaction between neural drive, spinal modulation, and muscular function determines the overall capacity to sustain performance under high-intensity conditions [148].

Ultimately, these neuromuscular processes translate into mechanical loading at the muscular level, which drives the intracellular adaptations described in subsequent sections. In this context, neural activation serves as the initial trigger for the mechanotransduction pathways that regulate muscle remodeling and performance adaptation.

However, the contribution of neuromuscular adaptations to high-intensity performance is highly context-dependent and influenced by factors such as exercise duration, fatigue mechanisms, and the relative contribution of metabolic versus neural limitations. While enhanced motor unit recruitment and firing frequency play a critical role in explosive and short-duration efforts, their relative importance may be reduced in prolonged or repeated high-intensity activities where metabolic constraints become more dominant.

Moreover, neuromuscular fatigue is not solely determined by peripheral mechanisms but is strongly influenced by central factors, including neural drive, motor cortex excitability, and psychological state. This highlights the need to consider neuromuscular function within an integrated framework that accounts for interactions between neural, metabolic, and psychological systems.

Therefore, optimizing high-intensity performance requires an integrated approach that aligns neuromuscular adaptations with metabolic demands and task-specific performance characteristics, rather than targeting these systems in isolation. As illustrated in Figure 3, neuromuscular fatigue emerges from dynamic interactions between central motor drive, spinal modulation, peripheral muscle mechanisms, and afferent feedback loops.

During repeated high-intensity exercise, increased neural drive recruits additional motor units, thereby elevating ATP demand and accelerating metabolic stress [85,149]. The resulting accumulation of metabolites modifies afferent feedback to the central nervous system, which subsequently alters motor output and movement mechanics [150]. These reciprocal interactions continuously reshape physiological adaptation, reinforcing that neuromuscular fatigue and performance emerge from an integrated biological network rather than isolated physiological mechanisms.

From a Complex Adaptive Systems perspective, no single biomarker is sufficient to characterize emergent fatigue during high-intensity exercise. Rather, fatigue should be evaluated through the integration of complementary physiological indicators, including metabolic biomarkers (blood lactate, inorganic phosphate, hydrogen ions, and phosphocreatine depletion) [97], neuromuscular measures (electromyographic activity and voluntary activation) [151], inflammatory biomarkers (interleukin-6 and tumor necrosis factor-α) [152], markers of muscle damage (creatine kinase and myoglobin), autonomic indicators such as heart rate variability, and endocrine responses including cortisol and the testosterone-to-cortisol ratio [153,154]. Collectively, these multilevel biomarkers provide a more comprehensive assessment of fatigue development, recovery status, and readiness for subsequent high-intensity exercise, consistent with the Complex Adaptive Systems framework.

## 6. Molecular and Cellular Mechanisms of Adaptation

From a Complex Adaptive Systems (CAS) perspective, molecular adaptation should be understood as an emergent consequence of continuous interactions among mechanical loading, metabolic stress, neural activation, and intracellular signaling networks. Rather than functioning as isolated biochemical pathways, these molecular processes dynamically interact through reciprocal feedback mechanisms that collectively regulate muscle remodeling, energy metabolism, and performance adaptation.

Building on the neuromuscular processes described in the previous section, adaptations to high-intensity exercise ultimately occur at the molecular and cellular levels through complex intracellular signaling pathways. These adaptations arise from the integrated interaction of mechanical, metabolic, and neural stimuli, which collectively regulate muscle remodeling, energy metabolism, and performance capacity.

### 6.1. Mechanotransduction and Muscle Remodeling

Mechanical loading generated during high-intensity exercise represents a primary stimulus for skeletal muscle adaptation [107]. Through the process of mechanotransduction, mechanical forces are converted into biochemical signals that activate intracellular pathways responsible for muscle growth and structural remodeling [155,156]. Mechanosensitive structures, including integrins and focal adhesion complexes, detect mechanical stress and initiate downstream signaling cascades, most notably the FAK–mTOR pathway, which plays a central role in promoting protein synthesis and muscle hypertrophy [157,158,159]. In addition, tension-sensitive pathways such as YAP/TAZ contribute to the regulation of gene expression by translocating to the nucleus and activating genes associated with muscle growth and remodeling [160,161]. Beyond hypertrophic adaptations, mechanotransduction also influences cytoskeletal organization and enhances muscle–tendon unit stiffness, thereby improving force transmission and mechanical efficiency [155,162]. Importantly, neural activation patterns discussed in the previous section determine the magnitude and distribution of mechanical loading across muscle fibers, highlighting the integrative relationship between neuromuscular regulation and molecular adaptation [35,107,162]. Rather than acting as an isolated mechanical signal, mechanical loading interacts continuously with neural activation, metabolic stress, and intracellular signaling networks. From a Complex Adaptive Systems perspective, mechanotransduction functions as an integrative process through which multiple physiological subsystems coordinate adaptive responses via reciprocal feedback mechanisms.

Mechanical loading activates key mechanotransduction pathways, including FAK, mTOR, and YAP/TAZ, which regulate protein synthesis and contribute to mitochondrial adaptations, redox signaling, and fiber-type-specific responses. These signaling pathways do not operate independently but continuously integrate mechanical, metabolic, and neural inputs, illustrating how molecular adaptation emerges from coordinated system-wide interactions rather than isolated biochemical events.

However, the extent and nature of mechanotransduction responses are highly dependent on the characteristics of the mechanical stimulus, including load magnitude, contraction type, and time under tension. For example, resistance training typically induces strong hypertrophic signaling through high mechanical tension, whereas high-intensity interval training may promote more moderate mechanical loading but greater metabolic stress.

Moreover, not all molecular signaling responses translate directly into meaningful performance improvements, as adaptations are influenced by the interaction between mechanical, metabolic, and neural stimuli. This highlights the importance of considering mechanotransduction within a broader, integrated framework rather than as an isolated pathway. Therefore, optimizing training-induced adaptations requires careful manipulation of loading strategies that balance mechanical, metabolic, and neural stimuli according to the athlete’s physiological state, allowing coordinated system-wide adaptation rather than isolated molecular responses.

### 6.2. Mitochondrial Function and Bioenergetic Adaptations

High-intensity training induces profound adaptations in mitochondrial structure and function, primarily through the activation of peroxisome proliferator-activated receptor gamma coactivator-1 alpha (PGC-1α), a key regulator of mitochondrial biogenesis [19,163]. From a Complex Adaptive Systems perspective, these mitochondrial adaptations should not be viewed as isolated bioenergetic responses but as components of an interconnected adaptive network that continuously interacts with neuromuscular regulation, metabolic stress, and redox signaling.

These adaptations enhance oxidative capacity, improve ATP resynthesis, and increase metabolic efficiency during repeated high-intensity efforts [164]. Increased mitochondrial density contributes to improved endurance capacity and accelerates recovery between exercise bouts [165]. Furthermore, mitochondrial adaptations facilitate the integration of aerobic and anaerobic energy systems, allowing for sustained performance under high metabolic demand [165,166]. High-intensity interval training (HIIT) is particularly effective in stimulating these mitochondrial responses, making it a critical component of performance optimization [167,168,169]. Rather than acting independently, mitochondrial adaptations both influence and are influenced by neural activation, fatigue regulation, and intracellular signaling pathways, illustrating reciprocal interactions that collectively shape performance adaptation.

However, the contribution of mitochondrial adaptations to high-intensity performance is context-dependent and varies according to exercise duration and the dominant energy system. While enhanced oxidative capacity supports recovery and repeated-effort performance, its direct contribution to short-duration, maximal-intensity efforts may be limited. Moreover, excessive emphasis on oxidative adaptations may not optimally support neuromuscular or power-oriented performance outcomes, particularly in sports requiring explosive force production. This underscores the importance of balancing metabolic and neuromuscular training stimuli to achieve optimal performance adaptations. Therefore, mitochondrial adaptations should be considered as one interconnected component within a broader adaptive network, where their contribution to performance depends on continuous interactions with neuromuscular, molecular, and metabolic systems rather than as a standalone determinant of high-intensity performance.

### 6.3. Reactive Oxygen Species (ROS) and Redox Signaling

Reactive oxygen species (ROS), traditionally considered harmful metabolic byproducts, are now recognized as essential signaling molecules involved in exercise-induced adaptations [23]. From a Complex Adaptive Systems perspective, ROS function as regulatory mediators that link metabolic stress, mitochondrial adaptation, and cellular remodeling through dynamic and reciprocal interactions rather than acting as isolated biochemical byproducts. During high-intensity exercise, transient increases in ROS activate redox-sensitive signaling pathways, including AMPK, MAPK, and NRF2, which regulate mitochondrial biogenesis, antioxidant defense, and metabolic function [23,170]. However, the adaptive role of ROS is highly dependent on balance. Excessive antioxidant supplementation may blunt these beneficial signaling processes by disrupting redox homeostasis, thereby attenuating training-induced adaptations [171].

However, the effects of ROS on performance and adaptation are highly dose-dependent. While moderate increases in ROS are essential for initiating beneficial signaling pathways, excessive oxidative stress may impair muscle function and accelerate fatigue. Furthermore, the interaction between ROS production and training intensity highlights the importance of context, as high-intensity exercise may induce both adaptive signaling and transient performance limitations due to oxidative stress. These responses illustrate the nonlinear nature of molecular adaptation, whereby similar oxidative stimuli may produce beneficial or maladaptive outcomes depending on the athlete’s physiological state and the interaction of ROS with other regulatory systems. Therefore, maintaining an optimal redox balance is critical for maximizing training adaptations, rather than simply minimizing oxidative stress through external interventions.

### 6.4. Metabolic Stress and Intracellular Signaling

Metabolic stress is a key driver of adaptation during high-intensity exercise [172]. From a Complex Adaptive Systems perspective, metabolic stress should be viewed as a dynamic regulatory signal that continuously interacts with mechanical loading, neural activation, and intracellular signaling networks rather than as an isolated physiological consequence of exercise.

The accumulation of metabolites such as lactate, hydrogen ions, and inorganic phosphate acts not only as a limiting factor for performance but also as a potent stimulus for intracellular signaling [173,174]. Lactate, in particular, has emerged as an important signaling molecule that influences gene expression, mitochondrial adaptations, and intercellular communication [173,174,175]. Additionally, metabolic stress interacts synergistically with mechanical loading to amplify adaptive responses, reinforcing the importance of integrated training stimuli [176]. These reciprocal interactions coordinate molecular and physiological adaptation across multiple interconnected systems rather than within isolated signaling pathways.

However, the role of metabolic stress in performance and adaptation is highly context-dependent. While moderate levels of metabolite accumulation contribute to adaptive signaling, excessive metabolic stress may impair force production and accelerate fatigue, thereby limiting performance during high-intensity efforts. Moreover, the effectiveness of metabolic stress as a training stimulus depends on its interaction with mechanical loading and neuromuscular activation. These adaptive responses also involve important trade-offs, as the same metabolic stimulus may enhance molecular adaptation or impair performance depending on recovery status, exercise intensity, and the athlete’s physiological state.

This highlights the importance of integrated training approaches that balance metabolic and mechanical stimuli to optimize adaptation. Therefore, metabolic stress should be viewed not only as a byproduct of fatigue but also as a regulated stimulus that must be carefully managed to maximize performance outcomes.

### 6.5. Integrated Molecular Response to High-Intensity Training

Importantly, molecular adaptations do not occur in isolation but emerge from the dynamic interaction between mechanical, metabolic, and neural stimuli [177]. Mechanical loading initiates mechanotransduction pathways, metabolic stress activates intracellular signaling cascades, and neural drive determines motor unit recruitment patterns that shape these stimuli [155,178,179]. These pathways continuously influence one another through reciprocal feedback interactions, allowing molecular adaptation to emerge from coordinated system-wide regulation rather than isolated signaling events. This coordinated interaction reflects the concept of skeletal muscle as a complex adaptive system [180]. As a result, training-induced adaptations include enhanced muscle remodeling, improved energy utilization, and increased resistance to fatigue [181]. This integrative perspective provides a comprehensive framework for understanding how high-intensity exercise translates into performance improvements.

However, the integration of these molecular responses is highly dependent on training design and individual variability. The relative contribution of mechanical, metabolic, and neural stimuli may differ based on exercise type, intensity, and athlete characteristics. Consequently, similar training stimuli may produce different adaptive outcomes depending on the athlete’s physiological state, recovery capacity, and the interaction among multiple regulatory pathways. This highlights the importance of individualized and context-specific training approaches that consider the interaction between multiple adaptive pathways. Therefore, high-intensity performance should be understood as an emergent property of coordinated molecular and physiological processes, rather than the result of isolated adaptations.

## 7. Ergogenic Aids and Performance Enhancement

Ergogenic aids represent a critical component in optimizing high-intensity exercise performance by modulating physiological, neuromuscular, and metabolic processes [5,49,182]. Rather than acting as isolated enhancers, these interventions interact with training-induced adaptations across multiple biological levels, influencing energy availability, fatigue resistance, and recovery capacity [56,170]. The effectiveness of ergogenic aids is highly context-dependent, varying according to training status, exercise modality, and individual responsiveness [183]. Therefore, their role should be understood within an integrated framework that aligns supplementation strategies with specific training demands.

### 7.1. Caffeine and Neural Activation

Caffeine is one of the most extensively studied ergogenic aids, primarily exerting its effects through central nervous system stimulation [143]. By antagonizing adenosine receptors, caffeine enhances neural drive, increases alertness, and reduces perceived exertion during high-intensity exercise [184,185]. These effects contribute to improved motor unit recruitment and sustained force production, particularly under fatigue conditions [143,186]. In addition to central mechanisms, caffeine may influence peripheral processes, including excitation–contraction coupling and calcium release, further supporting muscle performance [143,187,188]. Importantly, caffeine has been shown to enhance performance in repeated high-intensity efforts, where the ability to maintain neuromuscular output is critical [2,143,189]. However, individual variability in response to caffeine, influenced by genetic and habitual intake factors, highlights the importance of personalized dosing strategies [190]. Recent evidence also suggests that problematic caffeine consumption may coexist with other maladaptive behavioral patterns, reinforcing the importance of individualized and context-specific caffeine use strategies [191]. However, the ergogenic effects of caffeine are dose-dependent and may vary considerably between individuals, with excessive intake potentially leading to negative effects such as increased anxiety, impaired sleep, and reduced performance in certain contexts.

### 7.2. Creatine and ATP Resynthesis

Creatine supplementation plays a central role in enhancing high-intensity performance by increasing phosphocreatine (PCr) availability within skeletal muscle [192,193]. This directly supports rapid ATP resynthesis during short-duration, high-intensity efforts, thereby improving power output, sprint performance, and repeated-effort capacity [194]. Beyond its immediate bioenergetic effects, creatine may also contribute to long-term adaptations by promoting greater training volume and enhancing muscle hypertrophy [195]. Increased cellular hydration and activation of anabolic signaling pathways further support muscle remodeling and performance improvements [196]. As such, creatine serves as a key modulator of the bioenergetic system, particularly in activities characterized by explosive and repeated high-intensity demands [197]. However, the effectiveness of creatine may be limited in endurance-dominant activities, where oxidative metabolism plays a greater role than phosphagen-based energy systems.

### 7.3. Beta-Alanine and Buffering Capacity

Beta-alanine supplementation enhances intracellular buffering capacity through its role in increasing muscle carnosine concentrations [198,199]. Elevated carnosine levels improve the ability to buffer hydrogen ions (H+), thereby delaying the decline in muscle pH associated with high-intensity exercise [200,201]. This buffering effect is particularly beneficial in efforts lasting between 30 s and several minutes, where metabolic acidosis is a major contributor to fatigue [199,202]. By attenuating the impact of acid–base disturbances, beta-alanine supports sustained muscle contractility and delays performance decrements [202]. Consequently, it is especially relevant in sports requiring repeated high-intensity bouts with limited recovery. However, its effectiveness is highly dependent on exercise duration, with limited benefits observed in very short or prolonged endurance activities.

### 7.4. Nitrates and Oxygen Utilization

Dietary nitrates, commonly derived from sources such as beetroot juice, enhance exercise performance primarily through improvements in nitric oxide (NO) bioavailability [203]. Increased NO production promotes vasodilation, improves blood flow, and enhances oxygen delivery to working muscles [204].

At the muscular level, nitrates may improve mitochondrial efficiency by reducing the oxygen cost of ATP production, thereby enhancing exercise economy [205]. These effects are particularly relevant in high-intensity efforts where oxygen utilization and metabolic efficiency play a limiting role [164]. Additionally, nitrates may contribute to improved contractile function and reduced fatigue during repeated exercise bouts [206,207]. However, the ergogenic effects of nitrates may be less pronounced in highly trained athletes, potentially due to already optimized physiological systems.

### 7.5. Integrated Perspective on Ergogenic Aids

Importantly, ergogenic aids should not be considered as standalone performance enhancers but rather as modulators of the integrated physiological system underlying high-intensity performance [49,208]. Their effectiveness is maximized when aligned with specific training modalities and physiological demands [117,209]. For example, creatine supplementation may complement resistance and sprint training by enhancing ATP availability and power output, whereas beta-alanine supports metabolic adaptations associated with high-intensity interval training. Similarly, caffeine interacts with neuromuscular mechanisms to sustain performance under fatigue, while nitrates influence oxygen utilization and metabolic efficiency [143].

This integrated perspective reinforces the concept that performance adaptations emerge from the interaction between training stimuli and targeted nutritional strategies. Therefore, optimizing high-intensity performance requires a coordinated approach that considers the timing, dosage, and specificity of ergogenic aids in relation to the underlying physiological systems they influence.

However, the effectiveness of ergogenic aids is highly context-dependent and influenced by factors such as training status, exercise modality, dosage, and individual variability. Not all supplements produce consistent benefits across different performance contexts, and in some cases, their effects may be negligible or highly individualized. Moreover, the interaction between ergogenic aids and training-induced adaptations is complex, as supplementation may enhance, attenuate, or have no effect on underlying physiological processes depending on timing and dosage. Therefore, ergogenic aids should not be viewed as universal performance enhancers, but rather as targeted interventions that must be strategically integrated within a comprehensive training and nutrition framework.

As presented in Table 2, effective optimization of high-intensity performance depends on strategically aligning training modalities, multilevel physiological adaptations, and ergogenic interventions based on specific performance goals.

## 8. Practical Implications

From an applied perspective, optimizing high-intensity exercise performance requires an integrated approach that aligns training modalities, multilevel physiological adaptations, and nutritional strategies with specific performance goals. Because performance emerges from dynamic interactions among multiple physiological systems, relying on a single training method or isolated intervention is unlikely to produce optimal outcomes. Instead, training programs should be designed to reflect the functional and physiological demands of the sport while accounting for individual athlete characteristics and contextual factors.

For instance, athletes aiming to improve repeated sprint ability (RSA) may benefit from combining high-intensity interval training (HIIT) with sprint-specific training. Where appropriate, beta-alanine supplementation may also be considered as part of an individualized nutritional strategy, as it has the potential to enhance intramuscular buffering capacity and delay fatigue during repeated high-intensity efforts. However, the magnitude of these effects may vary according to individual responsiveness, training status, and the specific performance demands of the sport.

Similarly, for athletes seeking to improve maximal strength and power, resistance and plyometric training are generally regarded as key training modalities for enhancing neuromuscular recruitment, rate of force development (RFD), and mechanical efficiency. Creatine supplementation may also be considered where appropriate to support rapid adenosine triphosphate (ATP) resynthesis and repeated high-intensity efforts, although its effectiveness may differ according to individual characteristics, training status, and nutritional practices.

For activities requiring greater fatigue resistance and metabolic efficiency, concurrent training approaches may be integrated, where appropriate, with nutritional strategies such as dietary nitrate supplementation. These strategies may improve oxygen utilization and exercise economy in some athletes; however, their effectiveness is influenced by individual physiological characteristics, training status, sport-specific demands, and the broader training context.

Overall, the effectiveness of these strategies is highly context-dependent and influenced by multiple interacting factors, including training status, recovery capacity, nutritional practices, sport-specific demands, and individual responsiveness to training and supplementation. In addition, inappropriate manipulation of training load or excessive reliance on specific interventions may contribute to accumulated fatigue or suboptimal physiological adaptation. Therefore, optimizing high-intensity exercise performance is best supported through the dynamic integration of training, nutrition, and recovery within an individualized, evidence-informed, and goal-directed framework.

The principles summarized in Table 3 illustrate the practical implications of adopting a Complex Adaptive Systems perspective. Rather than providing prescriptive recommendations or simple decision rules for selecting training strategies or ergogenic aids, this framework is intended to support adaptive and evidence-informed decision-making based on the continuous interaction among training load, recovery status, nutritional support, neuromuscular readiness, metabolic regulation, and the athlete’s individual context. Accordingly, the practical implications presented in this review should be interpreted as a conceptual guide to support individualized and context-specific decision-making rather than as universal recommendations.

## 9. Future Research Directions

Future research should move beyond reductionist approaches and further explore high-intensity exercise performance through integrative and systems-based frameworks. Emerging technologies such as artificial intelligence (AI) and machine learning offer promising opportunities for developing personalized training prescriptions capable of adapting to individual physiological responses, training history, and performance goals.

In addition, wearable technologies and real-time monitoring systems may provide continuous assessment of physiological, neuromuscular, and recovery-related variables, enabling more precise regulation of training load and fatigue management. The growing field of network physiology also presents a valuable framework for understanding how multiple physiological systems interact dynamically during exercise and recovery, potentially offering novel insights into performance optimization.

At the molecular level, future studies should integrate multi-omics approaches, including genomics, transcriptomics, proteomics, and metabolomics, to better understand the biological mechanisms underlying training adaptations and interindividual variability in performance responses. Such approaches may facilitate the identification of biomarkers associated with adaptation, fatigue, and recovery.

Furthermore, research should investigate the interaction between ergogenic aids and training-induced adaptations using personalized strategies that account for genetic background, training status, and sport-specific demands. Understanding these interactions may improve the effectiveness of supplementation protocols and support individualized performance enhancement.

Future research should also investigate how individualized nutritional strategies, including carbohydrate periodization, nutrient timing, recovery nutrition, dietary nitrate-rich foods, omega-3 fatty acids, and gut microbiota-targeted interventions, interact with adaptive training processes. Integrating personalized nutrition with physiological monitoring and biomarker-guided training may enhance recovery, optimize training responsiveness, and improve long-term performance beyond the effects of conventional ergogenic supplements alone.

Overall, future investigations should prioritize multidisciplinary and longitudinal approaches that integrate physiological, molecular, neuromuscular, psychological, nutritional, and technological factors to advance a more comprehensive understanding of high-intensity exercise performance as a complex adaptive system.

## 10. Practical Nutritional Perspectives Beyond Conventional Ergogenic Supplements

While conventional ergogenic supplements such as caffeine, creatine, beta-alanine, and dietary nitrates remain valuable tools for enhancing high-intensity exercise performance, they should not be considered in isolation. From a Complex Adaptive Systems (CAS) perspective, nutritional support should be integrated with training load, recovery status, metabolic demands, and individual physiological characteristics to optimize adaptive responses rather than targeting single physiological mechanisms. Consequently, nutrition should be viewed as a dynamic regulator of system-wide interactions that influence bioenergetics, neuromuscular function, molecular signaling, immune regulation, and recovery.

Practical nutritional strategies should therefore extend beyond supplementation alone. Carbohydrate periodization should be aligned with training intensity and competition demands to optimize glycogen availability while preserving metabolic flexibility. Likewise, adequate daily protein intake distributed evenly across meals and combined with post-exercise protein ingestion may maximize muscle protein synthesis, tissue remodeling, and recovery. Appropriate hydration and electrolyte replacement remain essential for maintaining cardiovascular function, thermoregulation, and neuromuscular performance, particularly during repeated high-intensity exercise.

Dietary quality also represents an important but often overlooked component of performance optimization. Increased consumption of nitrate-rich vegetables (e.g., beetroot, spinach, and arugula), omega-3-rich foods, antioxidant-rich fruits and vegetables, and polyphenol-containing foods may support vascular function, recovery, mitochondrial adaptations, and the regulation of exercise-induced inflammation when incorporated within a balanced dietary pattern. Similarly, emerging evidence suggests that maintaining a healthy gut microbiota through dietary fiber, fermented foods, and appropriate probiotic interventions may influence immune function, nutrient utilization, and recovery capacity, thereby indirectly contributing to athletic performance.

Finally, future nutritional practice should move toward personalized nutrition guided by physiological monitoring, training history, biomarker profiling, and sport-specific demands. Integrating nutritional strategies with wearable technologies, metabolic biomarkers, and adaptive training models may enable individualized nutritional interventions that dynamically respond to changes in training load, recovery, and athlete readiness. Such an integrated approach is fully consistent with the Complex Adaptive Systems framework, in which optimal performance emerges from coordinated interactions among nutrition, training, recovery, molecular signaling, and neuromuscular regulation rather than from isolated nutritional interventions.

## 11. Conclusions

High-intensity exercise performance is best understood as an emergent property arising from the dynamic interaction between neuromuscular, metabolic, molecular, and biomechanical systems rather than the result of isolated physiological mechanisms. This review highlights that training modalities, including high-intensity interval training, resistance training, plyometric training, and their combined application, induce complementary and interdependent adaptations across multiple levels of biological organization.

At the neuromuscular level, performance is governed by central and peripheral mechanisms that regulate force production, coordination, and fatigue resistance. At the molecular and cellular level, adaptations are driven by integrated signaling pathways involving mechanotransduction, mitochondrial function, redox signaling, and metabolic stress, which collectively contribute to muscle remodeling and energy regulation. Importantly, these adaptations do not occur independently but are shaped by the interaction between mechanical loading, neural activation, and metabolic demands.

Furthermore, ergogenic aids act as modulators of this integrated system by targeting specific physiological pathways. However, their effectiveness is highly context-dependent and influenced by factors such as training status, exercise modality, dosage, and individual variability. Therefore, supplementation strategies should be carefully aligned with training demands rather than applied as universal performance enhancers.

Overall, this multilevel perspective emphasizes the importance of integrative and individualized approaches to optimize high-intensity performance. Future research should further explore the interactions between physiological systems and develop applied frameworks that bridge the gap between mechanistic insights and real-world training practices.

## Figures and Tables

**Table 1:** Comparison Between Conventional Exercise Physiology and the Complex Adaptive Systems Perspective.

Aspect	Conventional (Reductionist) Perspective	Complex Adaptive Systems (CAS) Perspective
**Primary focus**	Individual physiological mechanisms	Interactions among multiple physiological systems
**View of performance**	Sum of isolated physiological capacities	Emergent property of an integrated biological network
**Adaptation**	Linear response to training load	Nonlinear, context-dependent, and threshold-dependent
**Fatigue**	Consequence of central or peripheral limitations	Emergent regulatory process arising from system-wide interactions
**Training response**	Expected to be relatively predictable	Depends on athlete state, recovery, nutrition, training history, and environmental context
**Role of recovery**	Separate component of training	Integral regulator of system adaptation
**Role of nutrition**	Supports energy supply and recovery	Modulates molecular signaling and adaptive interactions across systems
**Decision-making**	Optimize isolated variables (e.g., VO_2_max, strength, lactate)	Optimize interactions among training, recovery, nutrition, neuromuscular readiness, and biomechanics
**Practical implication**	Standardized training prescription	Individualized and adaptive training strategies

**Table 2:** Decision-Oriented Framework Linking Training Modalities, Multilevel Adaptations, and Ergogenic Aids for High-Intensity Performance.

Performance Goal	Primary Training Strategy	Key Mechanisms	Recommended Ergogenic Aid	When to Use	Limitations
Maximal Strength/Power	Resistance + Plyometric	Neural drive, mTOR, RFD	Creatine, Caffeine	Pre-competition/strength phase	Limited metabolic adaptation
Repeated Sprint Ability	HIIT + Sprint Training	PCr resynthesis, buffering capacity	Beta-alanine, Caffeine	In-season/team sports	High fatigue load
Fatigue Resistance	HIIT + Concurrent Training	Mitochondrial biogenesis, metabolic efficiency	Nitrates, Beta-alanine	Mid-season/endurance block	May reduce peak power
Explosive Performance	Plyometric + Neural Training	Motor unit recruitment, SSC efficiency	Caffeine	Pre-competition	Limited endurance benefits
Recovery Optimization	Low-intensity + nutrition	Redox balance, mitochondrial recovery	Nitrates, antioxidants (careful)	Between sessions	Overuse may blunt adaptation

**Table 3:** Systems-Based Considerations for Optimizing High-Intensity Exercise Performance.

Systems Principle	Conventional Reductionist Approach	Complex Adaptive Systems Perspective	Practical Considerations
**Optimize interactions rather than isolated variables**	Focuses on maximizing a single physiological adaptation (e.g., VO_2_max or strength).	Performance emerges from coordinated interactions among neuromuscular, metabolic, biomechanical, and molecular systems.	Training programs may benefit from simultaneously considering multiple physiological adaptations rather than optimizing a single system in isolation.
**Adapt training according to athlete state**	Identical training is expected to produce similar responses.	Training responses depend on recovery status, fatigue, nutrition, training history, and physiological readiness.	Training load may be adjusted, when appropriate, according to recovery status, readiness, and performance monitoring.
**Recognize nonlinear adaptation**	Assumes proportional relationships between training load and adaptation.	Small changes in workload may produce disproportionately large improvements or maladaptive responses after physiological thresholds are exceeded.	Progressive manipulation of training load may support adaptation when interpreted alongside fatigue, recovery, and athlete response.
**Manage fatigue as an integrated system response**	Fatigue is viewed primarily as central or peripheral.	Fatigue emerges from interactions among neural regulation, metabolic stress, afferent feedback, biomechanical adjustments, and intracellular signaling.	Recovery strategies, nutritional support, and load management may be integrated to help regulate systemic fatigue according to the athlete’s needs.
**Use ergogenic aids as system modulators**	Supplements are used primarily to enhance isolated physiological functions.	Ergogenic aids interact with training-induced adaptations across multiple physiological systems.	Ergogenic aids may be considered according to the athlete’s physiological characteristics, training status, performance goals, sport-specific demands, and the available scientific evidence.
**Individualize training prescriptions**	Standardized programs are broadly applied.	Adaptation depends on individual physiological characteristics and environmental context.	Training and nutritional interventions should be individualized based on athlete-specific responses, contextual factors, and ongoing performance monitoring.

Note: The practical considerations presented in this table are intended as a conceptual guide rather than prescriptive recommendations. Because high-intensity exercise performance emerges from complex interactions among multiple physiological systems, training and ergogenic strategies should be individualized according to athlete characteristics, training status, performance goals, and sport-specific context.

## Data Availability

No new data were created or analyzed in this study. Data sharing is not applicable to this article.
